# Effects of the Chernobyl Disaster on Thyroid Cancer Incidence in Turkey after 22 Years

**DOI:** 10.5402/2011/257943

**Published:** 2011-12-15

**Authors:** Hasan Acar, Bahri Çakabay, Ferit Bayrak, Tülay Evrenkaya

**Affiliations:** ^1^Department of General Surgery, Large City Municipal Hospital, Ankara, Turkey; ^2^Department of Surgical Oncology, Ankara University School of Medicine, Turkey

## Abstract

*Background*. Separate studies involving people who survived atomic bombs have shown that the risk for cancer remains high after 40 years, compared with the risk in the general population. An elevated risk may also remain in regions of Turkey near the Chernobyl disaster. *Patients and Methods*. A multidisciplinary study conducted in 2008, 22 years after the Chernobyl disaster, examined the thyroid cancer incidence in Rize, a province of Turkey located on the shore of the middle Black Sea. Approximately 100,000 people were screened, and a fine-needle aspiration biopsy was performed in 89 patients. *Results*. Based on postoperative histopathological examinations, thyroid cancer was diagnosed in six of the 100,000 people screened. *Conclusion*. Given a thyroid cancer frequency of approximately 8 in 100,000 in the Turkish population, according to the Turkish Cancer Research Association, the rate in Rize reflects no increase in the thyroid cancer incidence 22 years after the Chernobyl disaster.

## 1. Introduction

The Chernobyl disaster on 26 April 1986 resulted in the release of substantial amounts of radioactive materials, including iodine, cesium, plutonium, and strontium radioisotopes. Although iodine-131 has a short half-life (8 days), people can rapidly receive internal exposure after breathing contaminated air or consuming contaminated milk and leafy vegetables. After entry into the body, iodine becomes concentrated in the thyroid gland. Radiation doses are usually higher in children than in adults owing to a higher intake of milk and dairy products by infants and children, the smaller size of their thyroid glands, and their higher metabolic rate [1].

Reports have linked the Chernobyl disaster to an increased incidence of thyroid cancer in children. During 1992–2000 in Belarus, Russia, and Ukraine, about 4,000 cases of thyroid cancer were diagnosed in children and adolescents aged 0–18 years, with about 3,000 of these occurring in children aged 0–14 years. The survival rate has been 98.8% for the 1152 cases of thyroid cancer diagnosed among Chernobyl children in Belarus during 1986–2002. Eight children died due to thyroid cancer progression, and six patients died from other causes. One patient in Russia died from thyroid cancer [1].

Thyroid radioactivity levels were measured in each person within 2 months of the accident. Each person was screened four times for thyroid cancer, beginning as early as 12 years after the disaster and continuing for 10 years [2].

As radioactive materials might have reached the Black Sea shores of Turkey, which is a region considerably distant from Chernobyl (approximately 1500 km), it is speculated that the cancer incidence in the region may remain high for a long time after the disaster. Here, we report on a multidisciplinary study conducted 22 years after the Chernobyl disaster that examined the thyroid cancer incidence in Rize, a province of Turkey located on the shore of the middle Black Sea.

## 2. Patients and Methods

We conducted this study on approximately 100,000 people in Rize province, located on the Black Sea shore of Turkey, where iodine deficiency-related goiter is common. An extensive screening was performed, and approximately 1,000 patients with goiter underwent detailed examinations. Patients with a goiter received laboratory and imaging analyses, and a fine-needle aspiration biopsy (FNAB) along with a cytological examination was performed in 89 patients. As a result, differential thyroid cancer was diagnosed in five patients, who then underwent a total thyroidectomy, and the findings in three patients were considered suspicious.

## 3. Results

The histopathological examination and FNAB results as well as the ages and genders of the patients are shown in Table 1.

The histopathological findings in all patients in which papillary thyroid cancer was diagnosed agreed with the FNAB results. The histopathological examination of three suspicious cases revealed follicular cancer in one case, Hashimoto thyroiditis in one case, and nodular colloidal goiter in one case (Table 1).

Of the patients with differential thyroid cancer, five were female and one was male, and the average age was 36 years. No postoperative complications were observed in any of the patients.

## 4. Discussion and Conclusion

The most significant contamination from the Chernobyl disaster affected the Republics of Belarus and Ukraine, as well as the western region of the Russian Federation [1]. Nikiforov et al. and Williams stated that the incidence of papillary thyroid carcinoma (PTC) in children recently reported in Belarus and Ukraine can, beyond a reasonable doubt, be attributed to exposure to fall-out radiation from Chernobyl [3, 4]. The largest increase in the PTC rate was observed among children, 0–14 years of age at the time of diagnosis, from high-exposure areas suggesting that a high prevalence of preexisting iodine deficiency in combination with the unique susceptibility of younger people might have contributed to the carcinogenic exposure of the thyroid [1].

External exposure to ionizing radiation produces DNA damage and increases the rates of cancers such as thyroid, lung, and breast cancers and leukemia [5]. The most dramatic effect on physical health resulting from exposure to fallout from the Chernobyl disaster has been an increased incidence of thyroid cancer [6, 7]. These thyroid tumors have a particularly high rate of rearranged during transfection (RET)/PTC rearrangements (57–67%, mainly PTC3) [6, 8, 9].

Radiation-induced PTC is associated with RET/PTC rearrangements. Hamatani et al. compared adult-onset PTC with RET/PTC rearrangements and with BRAF mutations in patients throughout Japan. More than 70% of adult-onset PTC in patients not exposed to radiation was associated with mutations in the BRAF gene [2]. The researchers analyzed the genetic profiles of participants in the Radiation Effects Research Foundation follow-up study, comparing the profiles of 50 cancer patients who had been exposed to atomic bomb radiation with those of 21 patients who had not been exposed: a greater radiation dose, a shorter elapsed time since radiation exposure, and a younger age at the time of exposure were three factors independently associated with the development of adult-onset PTC with RET alterations [2, 10].

Separate studies have shown that the risk for cancer in people who have survived an atomic bombing begins to decline after 30 years, but still remains high after 40 years compared with the risk in the general population [2].

An accurate assessment of the future health effects from the Chernobyl accident is not possible because of uncertainties with respect to dose exposures, current debates over the actions of radiation, and issues regarding the delayed consequences of atomic bomb exposure [11].

The incidence of thyroid cancer has increased markedly over a relatively short period of observation in all areas of the Republic of Belarus and among all age categories [12]. Between 1970 and 2001, the age-adjusted thyroid cancer incidence rates have increased from 0.4 per 100,000 to 3.5 per 100,000 among males (+775%) and from 0.8 per 100,000 to 16.2 per 100,000 among females (+1925%) in Belarus [12]. In the present study, all patients with papillary thyroid cancer were female and one patient with follicular thyroid cancer was male.

The relative increase among males (+1020%) and females (+3286%) in “high-exposure” areas exceeded the increases among males (+571%) and females (+250%) in “lower-exposure” areas of Belarus [12]. A distance of about 300 km in diameter is considered the high-risk region [11], whereas the distance from Chernobyl to the Black Sea shores of Turkey is about 1500 km. However, the combination of radiation exposure and iodine deficiency increased the risk for thyroid cancer by twofold among children and adolescents in Rize who were exposed to iodine-131 from Chernobyl, suggesting a modifying effect of iodine deficiency [13]. The relatively high incidence of iodine deficiency-related goiter in Rize might also have made local residents more sensitive to ionizing radiation.

Differential susceptibility to radiation-induced cancer could explain why only a minority of the population most heavily exposed to radiation following the Chernobyl disaster developed cancer. The possibility of using gene expression data to measure this susceptibility has a number of implications for research, medicine, and radioprotection [14].

According to the Turkish Cancer Research Association, the frequency of thyroid cancer in Turkey is 8 in 100,000. In the present study, approximately 100,000 people in Rize were screened and six cases of histopathologically diagnosed cancer were found. This rate is not significantly different from the rate of thyroid cancer in the general population of Turkey.

Thus, the Chernobyl disaster has had no effect on the thyroid cancer incidence in the Black Sea region of Turkey.

## Figures and Tables

**Table 1:** Ages, genders, and fine-needle aspiration biopsy (FNAB) and histopathological findings of eight patients.

Patient no.	Age (years)	Gender	FNAB result	Histopathological finding
1	23	F	Papillary cancer	Papillary cancer
2	33	F	Papillary cancer	Papillary cancer
3	35	F	Papillary cancer	Papillary cancer
4	39	F	Papillary cancer	Papillary cancer
5	42	F	Papillary cancer	Papillary cancer
6	43	M	Suspicious findings	Follicular cancer
7	32	F	Suspicious findings	Hashimoto thyroiditis
8	52	M	Suspicious findings	Nodular colloidal goiter

F: female; M: male.

## References

[1] (2006). *Health Effects of the Chernobyl Accident and Special Health Care Programmes, Report of the UN Chernobyl Forum, Expert Group “Health”*.

[2] Hamatani K, Eguchi H, Ito R (2008). RET/PTC rearrangements preferentially occurred in papillary thyroid cancer among atomic bomb survivors exposed to high radiation dose. *Cancer Research*.

[3] Nikiforov Y, Gnepp DR, Fagin JA (1996). Thyroid lesions in children and adolescents after the Chernobyl disaster: implications for the study of radiation tumorigenesis. *Journal of Clinical Endocrinology and Metabolism*.

[4] Williams D (1996). Thyroid cancer and the Chernobyl accident. *Journal of Clinical Endocrinology and Metabolism*.

[5] Ito T, Seyama T, Iwamoto KS (1994). Activated RET oncogene in thyroid cancers of children from areas contaminated by Chernobyl accident. *The Lancet*.

[6] Fugazzola L, Pilotti S, Pinchera A (1995). Oncogenic rearrangements of the RET proto-oncogene in papillary thyroid carcinomas from children exposed to the Chernobyl nuclear accident. *Cancer Research*.

[7] Williams ED (2006). Chernobyl and thyroid cancer. *Journal of Surgical Oncology*.

[8] Mahoney MC, Lawvere S, Falkner KL (2004). Thyroid cancer incidence trends in Belarus: examining the impact of Chernobyl. *International Journal of Epidemiology*.

[9] Shakhtarin VV, Tsyb AF, Stepanenko VF, Orlov MY, Kopecky KJ, Davis S (2003). Iodine deficiency, radiation dose, and the risk of thyroid cancer among children and adolescents in the Bryansk region of Russia following the Chernobyl power station accident. *International Journal of Epidemiology*.

[10] Ron E, Lubin JH, Shore RE (1995). Thyroid cancer after exposure to external radiation: a pooled analysis of seven studies. *Radiation Research*.

[11] Nikiforov YE (2006). Radiation-induced thyroid cancer: what we have learned from chernobyl. *Endocrine Pathology*.

[12] Baverstock K, Williams D (2006). The Chernobyl accident 20 years on: an assessment of the health consequences and the international response. *Environmental Health Perspectives*.

[13] Detours V, Versteyhe S, Dumont JE, Maenhaut C (2008). Gene expression profiles of post-Chernobyl thyroid cancers. *Current Opinion in Endocrinology, Diabetes and Obesity*.

[14] Zengi A, Karadeniz M, Erdogan M (2008). Does chernobyl accident have any effect on thyroid cancers in Turkey? A retrospective review of thyroid cancers from 1982 to 2006. *Endocrine Journal*.

